# Protective down-regulated states in the human brain: A possible lesson from COVID-19

**DOI:** 10.1073/pnas.2120221119

**Published:** 2022-11-07

**Authors:** Nicholas D. Schiff, Emery N. Brown

**Affiliations:** ^a^Department of Neuroscience, Feil Family Brain Mind Research Institute, Weill Cornell Medicine, New York, NY 10065;; ^b^Department of Brain and Cognitive Sciences, Institute for Medical Engineering and Science and Picower Institute for Learning and Memory, Massachusetts Institute of Technology, Cambridge, MA 02139;; ^c^Department of Anesthesia, Critical Care and Pain Medicine, Massachusetts General Hospital, Boston, MA 02114

**Keywords:** coma recovery, neurometabolic state, enhanced GABAergic state, burst suppression, COVID-19

## Abstract

The COVID-19 pandemic has created a large population of patients who are slow to recover consciousness following mechanical ventilation and sedation in the intensive care unit. Few clinical scenarios are comparable. Possible exceptions are the rare patients in post-cardiac arrest coma with minimal to no structural brain injuries who recovered cognitive and motor functions after prolonged delays. A common electroencephalogram (EEG) signature seen in these patients is burst suppression [8]. Biophysical modeling has shown that burst suppression is likely a signature of a neurometabolic state that preserves basic cellular function “during states of lowered energy availability.” These states likely act as a brain protective mechanism [9]. Similar EEG patterns are observed in the anoxia resistant painted turtle [24]. We present a conceptual analysis to interpret the brain state of COVID-19 patients suffering prolonged recovery of consciousness. We begin with the Ching model and integrate findings from other clinical scenarios and studies of the anoxia-tolerant physiology of the painted turtle. We postulate that prolonged recovery of consciousness in COVID-19 patients could reflect the effects of modest hypoxic injury to neurons and the unmasking of latent neuroprotective mechanisms in the human brain. This putative protective down-regulated state appears similar to that observed in the painted turtle and suggests new approaches to enhancing coma recovery [12].

## Significance

Late recovery of consciousness following intensive care unit treatment of COVID-19 is a frequent and puzzling phenomenon. Many of these patients showed no signs of structural brain injuries and enjoyed full cognitive function prior to their illnesses. Prolonged coma and recovery accompanied with hypoxia and markedly reduced brain metabolism are also present in certain patients after cardiac arrest and certain vertebrate animals like the painted turtle. The latter have evolved specialized physiological processes to preserve brain function during prolonged coma linked to loss of oxygenation. We postulate the existence of a protective down-regulated state in specific clinical scenarios in humans similar to that observed in anoxia-tolerant vertebrates. Induction of this state suggests new approaches to enhancing coma recovery.

Treatment for severe COVID-19 has required weeks, and in some cases months, of mechanical ventilation using multiple anesthetic regimes (1). The COVID-19 pandemic has created a large population of patients who are slow to recover consciousness following mechanical ventilation and sedation in intensive care units (2). In principle, these sedative regimens alone could produce COVID-19’s prolonged unconsciousness as anesthetic use for surgery is associated with postoperative neurocognitive disorders (3, 4).

The prolonged recovery of consciousness seen in some COVID-19 patients has multiple contributing factors. There is a severe oxygenation problem produced by an acute respiratory distress syndrome (ARDS) (2). Indeed, many severe COVID-19 patients require specific respiratory therapies such as pressure support ventilation, administration of inhaled nitric oxide, and proning—ventilating the patient in the face down position—to optimize oxygenation (1). Additional factors complicating ARDS can include a systemic inflammatory response, coagulopathy, and varying degrees of multiorgan system impairment, particularly renal and hepatic. In COVID-19 patients who are slow to recover, neuroimaging studies usually show that brain structures remain intact (5, 6). Despite the plausibility that sedation and these additional factors could collectively account for the prolonged recovery, recent findings demonstrate that only hypoxia is associated with these prolonged recoveries. Moreover, the more profound the hypoxia, the more prolonged the delay (7).

Few clinical scenarios are comparable to COVID-19. A possible exception is a recent study of patients remaining unconscious for 3–6 wk following post-cardiac arrest coma with limited or minimal evidence of structural brain injury (8). A common electroencephalogram (EEG) signature seen in the post-cardiac arrest patients was burst suppression (9). While EEG findings in COVID-19 are only just emerging, many COVID-19 patients show severe slowing and discontinuous, intermittent EEG patterns similar to burst suppression (10, 11).

A simple biophysical model has been proposed that offers a metabolic mechanism to explain burst suppression (9). It is hypothesized that burst suppression is likely a signature of a neurometabolic state that preserves basic cellular function “during states of lowered energy” and thereby acts as a brain protective mechanism (8, 9). The observation of burst suppression and similar discontinuous EEG patterns in certain COVID patients (6, 11), many of whom have experienced extended periods of hypoxia, could be consistent with Ching’s metabolic hypothesis for burst suppression as a marker of a neuroprotective state.

We present a conceptual analysis to interpret the brain state of COVID-19 patients suffering prolonged recovery of consciousness. Our analysis begins with the Ching model and integrates findings from other clinical scenarios and studies of anoxia-tolerant vertebrates, such as the painted turtle. We postulate that prolonged recovery of consciousness in COVID-19 patients could reflect the effects of modest hypoxic injuries to neurons and the unmasking of latent neuroprotective mechanisms in the human brain. These mechanisms could be analogous to a protective down-regulated state (PDS) observed in anoxia-tolerant vertebrates (12, 13).

## Burst Suppression: A Marker of Decreased Brain Metabolism

Burst suppression is a well-known EEG pattern during which isoelectric (flat) periods alternate with active periods (9). This pattern has four common defining characteristics. First, burst suppression can be synchronous with a common origin across the scalp or asynchronous (14). Second, the more inactivated the brain becomes, the longer the suppression periods. Third, burst suppression is quasi-periodic and operates on a longer time scale than other types of neural activity that are typically associated with brain inactivation such as slow-delta oscillations (14). Whereas slow-delta oscillations can range from 1/4 to 2 s, the quasi-periodicity of burst suppression can range from 10 to 20 s (9, 15). Finally, the brain activity that was present prior to the onset of burst suppression is preserved within the burst periods (8, 9). These characteristics suggest that burst suppression can be a dynamic process affecting nearly the entire cortex.

Burst suppression is commonly seen during hypoxic brain injuries, hypothermia, high doses of GABAergic anesthetics, and Ohtahara syndrome, a developmental syndrome associated with cortical abnormalities and difficult-to-control seizures (16). Each of these conditions is associated with decreased cerebral metabolic rate of oxygen consumption (CMRO_2_). For hypoxic brain injuries and hypothermia, there is likely a direct effect on cerebral metabolism linked to reduced synaptic activity resulting from impaired neuronal and mitochondrial function. For the GABAergic anesthetics—propofol, the barbiturates, and the inhaled ethers—the decrease in CMRO_2_ is likely mediated through direct inhibition of adenosine triphosphate (ATP) synthesis and blocking of the respiratory chain within the mitochondria (17–19). For Ohtahara syndrome, the poorly organized cortical circuits generate vigorous seizure activity, and burst suppression likely shuts down this activity (16). Seizure activity followed by burst suppression is also seen in prolonged unconsciousness following cardiac arrest (8).

## Ching Model of Burst Suppression

Ching and colleagues (9) developed a biophysically based mathematical model that captures the essential features of burst suppression. At the heart of the model is a cortical network of inhibitory and excitatory Hodgkin Huxley neurons each defined by membrane, synaptic and applied currents. The metabolic state of the network is described by three ancillary equations: one defining the adenosine triphosphate-gated potassium (ATP-K) current; and two equations that describe the exchange of sodium and ATP. The relation to CMRO_2_ is modeled as the production rate constant of ATP. A decrease (increase) in the ATP production rate represents a decrease (increase) in CMRO_2_. The key feature of this system is that a decrease in ATP yields increases in potassium conductance and outward potassium currents due to inability to maintain the sodium-potassium-ATP-dependent pumps. Hence, neuronal membranes hyperpolarize, spiking activity shuts down, and the onset of the suppression period begins. The duration of the suppression period is proportional to the decrease in ATP, the free variable in this model. At the point where the potassium conductance reaches a maximum, ATP begins to recover, the potassium leak current terminates, the suppression period terminates, and the burst activity returns.

The Ching model without the metabolic component was specifically designed to produce the alpha oscillations (8–12 Hz) that are typically observed during unconsciousness maintained by GABAergic anesthetics (9, 20). The model shows that the alpha oscillations are preserved during the burst periods. In addition, the model describes: burst suppression’s quasi-periodic behavior; its slow time scale relative to other oscillations associated with brain inactivation; and the lengthening of the suppression periods with decreasing energy levels.

## Cognitive Recovery following Prolonged Coma in Post-Cardiac Arrest Patients

Forgacs and colleagues (8) used the Ching model to interpret the recovery after prolonged coma following cardiac arrest of three patients treated with controlled hypothermia. The patients had limited or minimal evidence of structural brain injuries and remained in a coma for 3–6 wk. All achieved excellent cognitive and physical recoveries. In each patient, coma outlasted sedation and the EEG showed burst suppression. There was a dominant 7 Hz rhythm within the burst activity (8, 15). In a prospective study of 53 additional patients, Forgacs and colleagues (8) found a positive correlation between the presence of the 7 Hz spectral peak in the intraburst activity and favorable cognitive outcomes. The use of therapeutic hypothermia likely played a key mechanistic role in decreasing CMRO_2_, and thereby, contributing to a neuroprotective state (8). Hence, the report by Forgacs and colleagues (8) supports the Ching hypothesis that burst suppression is likely a marker of a protective state.

## Trace Alternant and the Anoxia-Tolerant Painted Turtle

The Ching model for burst suppression provides a general mechanism for slowing (9) or shutting down brain activity by lowering brain energy use through sedation with GABAergic agents. In this model, membrane hyperpolarization is controlled by the rate of ATP formation. In turn, this variable can be sharply down-regulated by mechanisms independent of sedation, particularly deafferentation (i.e., loss of afferent cortical pathways), as may occur in severe anoxic injury. Cunningham and colleagues (21) developed an earlier model demonstrating overall control of the dynamics of the EEG by K-ATP manipulations. This linked metabolism to slow and fast network dynamics present across the full range of arousal levels and pathological states of seizures. Indeed, Ching and colleagues (9) also noted that burst suppression may reflect a more general strategy of neuroprotection in the setting of sharp reduction of metabolic substrate. They further proposed that the “trace alternant” EEG pattern, observed in neonates, bears a strong relationship to burst suppression, and could reflect a parallel strategy for preserving the integrity of cortical neurons, by limiting excess or runaway excitation. Trace alternant is a typical sleep pattern in term neonates and could reflect an energy conservation state (22).

An EEG pattern that is almost identical to trace alternant has been observed in the painted turtle, perhaps nature’s most anoxia-resistant vertebrate (23). During the winter, this turtle faces severe anoxic conditions and exhibits a marked temperature-dependent neuroprotective response. For temperatures near 20 ^∘^C, the turtle can withstand 48 h of anoxia, whereas for temperatures near 1^∘^ to 3 ^∘^C, the animal can withstand 5 mo of persistent anoxia. Following reoxygenation, normal neuronal function returns within hours (23). During anoxia, the turtle’s trace alterant patterns consist of bursts of mixed frequency activity that occur at intervals of 0.5–2 min (24). A key mechanism for long-term maintenance of this state in the turtle involves GABA-mediated suppression of spontaneous neuronal spiking activity and synaptic transmission (25). After 2 h of anoxia, extracellular GABA levels in the painted turtle increase 80-fold (26). This GABA release, termed the “endogenous anesthesia for the anoxic turtle brain” (12), most certainly contributes to both neuronal and mitochondrial shutdown.

To explain brain adaptations to hypoxia/anoxia and hypothermia in anoxia tolerant vertebrates, Hochacka and Guppy (27) proposed a general framework that consists of: initial ion channel arrest and rapid reduction of metabolic demand; a drop in overall metabolic rate and failure of ATP-dependent maintenance of membranes leading to intracellular changes; and protection of intracellular structures from damage or denaturation triggered by the altered intracellular milieu. In addition, vertebrates like the painted turtle have evolved a variety of complementary strategies (28–30). These include alterations in patterns of gene expression (31), new protein synthesis (32), phosphorylation/dephosphorylation of regulatory proteins (29), and a broad collection of other molecular mechanisms (12, 13, 33).

## Direct Neuronal Injury in Response to Hypoxia

The frequent hypoxia characteristic of COVID-19 patients likely contributes to neuronal injury rather than cell death (1, 34). For example, it has been shown in experimental preparations that in the absence of ischemia, the brain tolerates hypoxia well (35). However, during non-ischemic hypoxia normal neuronal functions, such as stimulus encoding (36) and information integration (37), can be impaired. Hypoxic injury may also result specifically in sequestering or “quarantining” of mitochondria within pyramidal cells in cortical layer V (38). Such a process could affect the output of cortical columns and potentially alter level of consciousness. Furthermore, hypoxia-related impairment of neuronal function may be exacerbated by concomitant pathologic conditions such as hypercarbia and hyponatremia (39, 40). Despite the likelihood of hypoxia-related neuronal injury, the prolonged recovery of consciousness observed in many COVID-19 patients following termination of mechanical ventilation (5–7) suggests the possible protective effects of reduced neuronal activity. This hypothesis is supported by the Ching model, the observations made by Forgacs and colleagues (8) in post-cardiac arrest patients and the known physiology of anoxia resistance in the painted turtle.

## Does the Prolonged State of Impaired Consciousness after Severe COVID-19 Treatment Reflect a Protective Down-Regulated State?

As noted, certain vertebrate species, like the painted turtle, have evolved neuroprotective mechanisms to withstand extended periods of metabolic stress (41–44). These mechanisms support the concept of a protective down-regulated state (PDS) for the brain. Vertebrate PDS mechanisms have evolved to reduce brain energy demands when systemic metabolic stresses, such as anoxia or temperature extremes overwhelm the organism (28, 29). PDS may have unrecognized correlates in the human brain (41).

We propose that hypoxia combined with certain therapeutic maneuvers (28, 29, 43–46) may initiate an as yet unrecognized PDS in humans that results in prolonged recovery of consciousness in severe COVID-19 patients following cessation of mechanical ventilation (5–7) and in post-cardiac arrest patients treated with hypothermia (8). In severe COVID-19 patients, we postulate that the specific combination of intermittent hypoxia, severe metabolic stress, and GABA-mediated sedation may provide a trigger for the PDS. Analogous conditions are also common in post-cardiac arrest coma and in the painted turtle’s anoxia-induced coma (Table 1).

**Table 1:** Characteristics of actual and putative protective down-regulated states

	Painted turtle	Post-cardiac arrest patients	Post-COVID-19 patients
Brain state	Structurally intact and initially healthy	Structurally intact and initially healthy	Structurally intact and initially healthy
Metabolic state	Extreme hypothermia	Therapeutic hypothermia to 33 degrees for 24 h with rewarming over 24 h	Extreme systemic stress and metabolic demands due to viral infection and possible multiorgan system dysfunction
Oxygenation state	Profound anoxia	Severe anoxia for 10–20 min	Intermittent hypoxia
GABAergic effects	Endogenous sedation through GABAergic mechanisms	Prolonged deep sedation with GABAergic agents	Prolonged deep sedation with GABAergic agents
EEG patterns	Trace alternants with burst suppression	Burst suppression	Slow-wave oscillations

The key common factor in establishing a PDS in vertebrates is deep global suppression of cerebral metabolism (47, 48) initiated by endogenous GABAergic mechanisms. In COVID-19 patients who exhibit prolonged recovery of consciousness, deep global suppression is likely achieved by sedation with GABAergic anesthetics in intact healthy brains. However, mechanical ventilation with sedation mediated by GABAergic anesthetics alone is unlikely to provide a sufficient stimulus to engage a PDS without hypoxia initiating the process (49). In the painted turtle, once hypoxic and hypothermic conditions ensue, there appear to be two mechanisms overlapping those of GABAergic anesthesia that initiate a PDS: 1) activation of K-ATP channels (50); and 2) intense and specialized engagement of GABAergic networks (25). We propose that the use of GABAergic anesthetics may thus play a crucial causal role in unmasking a human PDS through conserved mechanisms to suppress excitatory neural activity using GABAergic networks (25). In the painted turtle, concomitant with anoxia and hypothermia, there is an 80-fold increase in GABA production and a 20-fold increase GABA sensitivity (51). In anoxia tolerant vertebrates, specific systems sense hypoxia and initiate anoxia-mediated spike arrest through GABAergic mechanisms that show burst suppression (48). Activation of these or similar mechanisms in humans by GABAergic anesthetics likely explains the known selectivity of these agents for producing burst suppression (4). The Ching model provides a general mechanism to shut down brain activity in low-energy settings of deep sedation. Similarly, the K-ATP channels play an important role in the painted turtle’s mechanism to protect the brain. Prior to achieving the protected state, K-ATP channel activation is known to produce marked slowing of neuronal population activity (21) (Table 2).

**Table 2:** Hypothesis for COVID-19 protective down-regulation state

1. A severe prolonged COVID 19 infection produces extreme metabolic demands, hypoxia, and a global scarcity of ATP.
2. Aggressive intensive care management often requires sedation with heavy doses of GABAergic anesthetics to maintain adequate oxygenation.
3. In certain COVID-19 patients, hypoxia cannot be fully rectified by optimization of mechanical ventilation. The ensuing ATP scarcity leads to opening of neuronal K-ATP channels and a global shut-down of brain activity, likely marked by burst suppression.
4. The combination of global ATP scarcity and heavy sedation with GABAergic anesthetics likely helps initiate a protective down-regulation state.

Use of hypothermia as a therapy following cardiac arrest (8) likely provides a powerful, synergistic factor for inducing PDS, as hypothermic conditions drive many of the evolved vertebrate PDS mechanisms (42). Intraneuronal signals that initiate a PDS through transcriptional changes that alter excitatory and inhibitory neurotransmitter activity have been identified in response to hypothermia within painted turtle neurons (52). If a human PDS underlies the protective effects of therapeutic hypothermia or targeted temperature management, it would help explain the inefficacy of these strategies in traumatic brain injuries (TBI) (53). That is, the damaged neuronal networks created by brain trauma may not be able to execute the protective programs that hypothermia would initiate in intact neurons exposed in anoxia or hypoxia. However, induction of burst suppression alone does appear to be associated with improved outcomes in severe TBI (54).

If prolonged recovery of consciousness in COVID-19 patients does reflect a human PDS, several measurements may be informative. First, in other vertebrates, changes in excitatory and inhibitory neurotransmitters and receptors occur immediately in response to hypoxia associated with the spike arrest phase of the PDS response (44, 55). (Table 3) Second, gene expression for GABA-A receptors increases as part of the sharp rise in GABA activity present during the painted turtle’s protective coma (51). Third, after the painted turtle spends months in protective coma, a rapid recovery phase ensues without damage from reactive oxygen species during reperfusion. This phase is associated with increases in neuroglobin protein (Ngb), a heme protein thought to catalyze redox activities that protect cells against fluctuations in oxygen level (56). Human Ngb exists (57) and can be measured in cerebrospinal fluid (58). Such a role for human Ngb has not been established, but direct measurement of elevated human Ngb would support the proposed PDS mechanism. It could act as a cytoprotective agent during reoxygenation given its role in preserving neuronal function during PDS in the painted turtle and in the crucian carp (59, 60). Finally, in addition, changes in adenosine, heat shock proteins and other factors associated with PDS in different vertebrates (45, 59, 60) may inform our understanding of possible human PDS mechanisms.

**Table 3:** Strategies to facilitate emergence from putative COVID-19 PDS

ATP support via Szeto-Schiller peptides (compensation for need to shut down neuronal membrane via K-ATP channel) to restore cellular energy resources
Amantadine to increase frontostriatal excitatory neurotransmission via NMDA antagonism (reverses the broad excitatory down-regulation)
Donezepil, a cholinergic agonist, to facilitate long-range excitatory neurotransmission at the level of dendrosomatic coupling within cortical Layer V pyramidal neurons

## Managing Prolonged Recovery of Consciousness following COVID-19

If a human PDS underlies prolonged recovery of consciousness in severe COVID-19 patients, we propose a two -part strategy to promote emergence (Table 3). In all anoxia-tolerant animals, maintenance of brain ATP when exposed to anoxia is critical for preventing neuronal death (30). Mitochondrial dysfunction is expected to accompany hypoxia and inflammation in severe COVID-19 (59). Thus, first improving mitochondrial ATP production may have a specific benefit in COVID-19 therapy. For this reason, Szeto-Schiller peptides, which are currently under clinical investigation for a variety of indications, should be considered (61–63). These small, four-amino acid peptides restore mitochondrial ATP function in aged rodents to that of young adult within an hour of administration (64). They cross the blood-brain-barrier (65) and they prevent loss of tubular structure in acute tubular necrosis (66). The peptides intercalate into mitochondrial membranes and act through electrostatic forces to restore membrane curvature and facilitate electron transport (63). For this reason, they can be termed geometroceuticals. Increasing ATP production could help mitigate post-hypoxic neuronal injury, meet the demands of increased metabolism induced by inflammation (59) and support high energy requirements of excitable cell (65). In particular, increasing ATP would support a variety of high energy intracellular and intercellular functions including those important for modulating synaptic function (67, 68) and integrating multiple inputs at synaptic sites (36, 37). Moreover, direct ATP support could directly mitigate mitochondrial dysfunction produced by sedation with GABAergic anesthetics (18).

As a second part, pharmacological agents used to treat cognitive impairment following disorders of consciousness (2, 34, 66) could be combined with direct ATP therapy for treatment of COVID-19 patients. Post COVID-19 neuropsychological deficits (69) overlap those of ARDS, mixed intensive care unit (ICU) cognitive impairments, and those seen in patients with moderate to severe TBI (70). All of these forms of cognitive impairment reflect fronto-striatal neuronal dysfunction (69, 70). Combining glutamatergic support using amantadine, the first-line drug for treating disorders of consciousness resulting from TBI (71–74) with the cholinergic agonist donepezil would be a novel therapeutic approach. Increasing glutamatergic tone would likely drive frontal cortical and striatal neurons (69–72), whereas increasing cholinergic tone may improve intracellular function in cortical Layer V cells (75). Uncoupling of Layer V outputs from incoming dendritic inputs has been recently shown to occur through blockade of glutamatergic and cholinergic receptors by anesthetics (75).

## Conclusions

The natural history of recovery of consciousness of COVID-19 patients after ICU care is being documented. Our analysis predicts the existence of a human form of PDS that may underlie prolonged recovery of consciousness following treatment for severe COVID-19 or treatment for post-cardiac arrest treated with hypothermia. The possible existence of a human PDS suggests many testable hypotheses for further investigation and the possibility of developing novel therapeutic strategies. These directions should be guided by the elements of successful rescue of human brains from pathological states and the recovery of other vertebrate brains from extreme conditions. The putative human form of PDS suggests that negative prognostic conclusions should be strictly avoided in any patients without brain injuries, independent of duration of impaired consciousness (2, 5, 8).

## Data Availability

All study data are included in the article.
